# Predictors for Adolescent Visits to Practitioners of Complementary and Alternative Medicine in a Total Population (the Young-HUNT Studies)

**DOI:** 10.1371/journal.pone.0025719

**Published:** 2011-10-07

**Authors:** Aslak Steinsbekk, Marit By Rise, Felicity Bishop, George Lewith

**Affiliations:** 1 Department of Public Health and General Practice, Norwegian University of Science and Technology, Trondheim, Norway; 2 Complementary and Integrated Medicine Research Unit, Primary Medical Care, University of Southampton, Southampton, United Kingdom; The University of Queensland, Australia

## Abstract

**Aim:**

To investigate the factors predicting adolescent visits to practitioners of complementary and alternative medicine (CAM).

**Methods:**

A longitudinal cohort study conducted in an adolescent total population in Central Norway (The Nord-Trøndelag Health Studies (HUNT)). In Young-HUNT 1, all inhabitants aged 13 to 19 years (N = 8944, 89% response rate) were invited to participate, and the youngest group (13 to 15 year olds) was surveyed again 4 years later (Young-HUNT 2, N = 2429, 82% response rate). The participants completed a comprehensive questionnaire on health and life style which included a question regarding visits to a CAM practitioner in the last 12 months.

**Results:**

One in eleven (8.7%, 95%CI 7.6-9.8%) had visited a CAM practitioner, an increase of 26% in 4 years (1.8% points). The final multivariable analysis predicted increased odds of an adolescent becoming a CAM visitor four years later (p<0.05) if she or he had previously visited a CAM practitioner (adjOR 3.4), had musculoskeletal pain (adjOR 1.5), had migraine (adjOR 2.3), used asthma medicines (adjOR 1.8) or suffered from another disease lasting more than three months (adjOR 2.1). Being male predicted reduced odds of visiting a CAM practitioner in the future (adjOR 0.6).

**Conclusion:**

We can conclude from this study that future visits to a CAM practitioner are predicted by both predisposing factors (being female, having visited a CAM practitioner previously) and medical need factors (having had musculoskeletal pain, migraine, used asthma medicines or experienced another disease lasting more than three months). None of the specific variables associated with CAM visits were predictive for CAM visits four years later.

## Introduction

Most research on the utilisation of complementary and alternative medicine (CAM) has focused on the adult population [1]. Nevertheless, a sizable proportion of those using CAM are children and adolescents [2], [3], with one in eight children using CAM in the USA in 2007 [2] and one in six using CAM among the adolescent population (12 to 17 years). CAM use includes both self medication and visit to CAM practitioners [4]. It is estimated that the paediatric population in the USA used 127 million dollars visiting CAM practitioners in 1996 [5].

Most studies on CAM consumption in adolescents are based on cross sectional studies [6]. These provide valuable insights into the factors that are associated with CAM use, but have limited value in identifying whether such factors contribute to the initiation of CAM use, are consequences of CAM use, or are not directly causally related to CAM use at all. Longitudinal studies can identify the factors that predict CAM utilisation, and can thus aid the interpretation of cross-sectional studies. We have only been able to identify one longitudinal study of CAM use in adolescents, which surveyed parents of 182 adolescents with juvenile idiopathic arthritis [7].

The socio-behavioural model of health services use [8], [9] (SBM) posits that healthcare utilisation is determined by three classes of variables: societal determinants, health service system features, and individual determinants. Individual determinants have received the most attention in the CAM literature, and can be thought of as predisposing factors (e.g. demographic characteristics), enabling factors (e.g. availability of services), and medical need (e.g. perceived health status). This model has been used to understand CAM use [10], [11] and has recently been identified in a systematic review as a particularly promising framework for research in this area [12].

The aim of this study was to identify the factors predicting adolescent visits to CAM practitioners and to explore the difference between variables predictive of future CAM visits and the association between the same variables and CAM visits in a cross sectional study.

## Methods

This was a longitudinal study with data from The Nord-Trøndelag Health Studies (HUNT, http://www.ntnu.no/hunt/english) involving the youth cohort (Young-HUNT 1 and 2). HUNT is one of the largest health studies ever performed involving the personal and family medical histories of 120,000 people from Nord-Trøndelag county in Central Norway. Nord-Trøndelag is one of 19 counties in Norway and has a stable and homogenous population of nearly 130,000 people with approximately 10% being in the age group 13 to 19 years. It is very similar to Norway as a whole in most demographic variables including sex and gender distribution, economy, and source of income and employment [13]. There are no large cities and the average income and education level is somewhat lower than in the rest of Norway.

### Ethics Statement

A written consent to take part in the study was signed by both a parent and the participant if the participant was under the age of 16 years. Participants 16 years or older were legally able to provide consent without additional consent from a parent/guardian. The study was approved by the Regional Committee for Medical Research Ethics and the Norwegian Data Inspectorate Board.

### Participants

Young-HUNT 1 was conducted from August 1995 to June 1997 and the target population was those aged 13 to 19 years (school year 8 to 13). The study was conducted in schools and the list of pupils was the main source for the invitation to participate. The invitation was sent to 9,917 adolescents and 8,944 (90%) participated. The survey was conducted during one school hour in an exam setting where it was not possible to see the response of the other participants. They answered more than 100 questions and also completed a clinical exam within one month (clinical data not used here).

Young-HUNT 2 was conducted four years later from January 2000 to June 2001. Adolescents in school years 12 and 13 and those in apprenticeships (school years 8 to 10 in Young-HUNT 1) were targeted. A total of 2,969 participants in Young-HUNT 1 were eligible and invited to participate in Young-HUNT 2. The survey was conducted in exactly the same way as Young-HUNT 1.

Those participating in both Young-HUNT 1 and 2 were included in this study.

### Measures

The HUNT survey included items that can be reconceptualised in terms of the SBM, although no measures of enabling factors were available.

Health services utilisation was measured for the dependent variable and for use of conventional health service. A CAM visitor was defined as anyone answering yes to: “During the last 12 months, have you been to a: Homeopath/Other treatment-provider such as naturopath, reflexologist, layer on of hands, healer, visionary, or corresponding service?” (Yes/No). Conventional health service use was measured with questions on visits to a physician or psychologist during the last 12 months. “Visit to a physician” was determined by participants answering “Yes” to having visited a general practitioner, a doctor at a hospital without being admitted, or being admitted to a hospital.

The predisposing factors were items that assessed socio-demographic characteristics (age, sex) and three lifestyle factors: daily smoking “Do you smoke” (No  =  no, previously or occasionally/Yes  =  daily); active in sports “Are you actively involved in sports? (No  =  no or was before/Yes); intoxication “Have you ever drunk so much alcohol that you felt intoxicated?” (No  =  never/Yes  =  once or more).

Several measures of medical need were used (translated from Norwegian).

- Self reported Global Health: “How is your health at the moment?” (Very good/Good/Fair/Poor).
- Limitation due to physical or mental health: “Are you functionally disabled in any way? Impairment due to physical illness/mental health complaints” (No  =  no/Yes  =  a little, somewhat or severely).
- Content with life: “Thinking about your life at the moment, would you say you overall are satisfied with life or are you mostly dissatisfied?” (No  =  very dissatisfied, dissatisfied or somewhat dissatisfied/Yes  =  Very satisfied, satisfied or somewhat satisfied).
- Lonely: “Do you feel lonely?” (No  =  very seldom or never, seldom, sometimes/Yes  =  often, very often).
- Recent health complaints: “Have you had any of these ailments in the past 12 months? Headache, neck or shoulder pain, joint or muscle pain, stomach pain, nausea, constipation, diarrhoea, heart palpitation” (No  =  Never/Yes  =  Seldom, Sometimes or Often), “Have you in the past 12 months had wheezing or whistling in the chest/itchy rash/sneezing, runny or blocked nose when you did not have a cold or the flu/these nose problems accompanied by itchy-watery eyes?” (No/Yes).
- Diseases: “Have you had any of these diseases in the past 12 months? Bronchitis or pneumonia, ear infection, sinus infection” (No  =  Never/Yes  =  Seldom, Sometimes, or Often)”, “Have you ever had hay fever or nose allergies?” (No/Yes), “Have you ever had eczema?” (No/Yes), “Has a medical doctor said that you have Asthma, Epilepsy, Diabetes, or Migraines?” (No/Yes), “Have you had any other diseases that lasted more than three months?” (later regrouped into heart disease, lung disease, kidney disease, abdominal disease, musculoskeletal disease, rheumatism, cancer, allergies, cerebral palsy, neurological disease, mononucleosis, other) (No/Yes).
- Medicines: “Do you take any of these medicines? Pain relievers, migraine medicine, sleep medicine, nerve medicine, relaxants, asthma medicine, allergy medicine, eczema cream” (No/Yes).

### Analysis

First, adolescents who reported visiting a CAM practitioner were compared with those who had not using a Pearson chi-square test. In the subsequent analysis, multivariable logistic regression was used to calculate adjusted odds ratio (adj OR) by controlling for all variables in the models. The same analysis was used for both the cross sectional (association between CAM visits and other variables in YH1) and the longitudinal analysis (variables in YH1 predicting CAM visits in YH2). The precision of the prediction is indicated by a 95% confidence interval (95% CI). To make it easier to identify the variables most strongly associated with visits to a CAM practitioner, the variables with a significance level below 5% (p<0.05) are marked in the tables. All data was analyzed using SPSS, version 17.0 for Windows (SPSS Inc., Chicago, IL, USA).

## Results

Of the 2,969 eligible younger adolescents who participated in Young-HUNT (YH1, 1997), 2,429 (81.8%) also participated in Young-HUNT 2 (YH2, 2001). Their average age in YH1 was 14 years and 53.4% were females. Nearly one in four had felt lonely often or very often while 14% were not content with their life. They reported having on average 4.2 (median 4, inter quartile range (IQR) 2-6) recent complaints, 1.2 (median 1, IQR 0-2) diseases and used 1.1 (median 1, IQR 0-2) types of medicines. One half (52%) had visited a physician within the last year and 92.3% had good or very good health.

### Prevalence of CAM visits

One in fourteen (6.9%, 95% CI 5.9–7.9%) had visited a CAM practitioner during the last year at baseline. In YH2, one in eleven (8.7%, 95% CI 7.6-9.8%) had visited a CAM practitioner, an increase of 26% (1.8% points) in 4 years (p<0.001). A total of 13.9% of the adolescents had visited a CAM practitioner in either YH1 or in YH2. Of those who visited a CAM practitioner in YH2, 19.0% also visited in YH1.

### Predictors for CAM visits

Table 1 shows the variables in YH1 that had a statistically significant bivariate prediction with visits to CAM practitioners in YH2 four years later (table 1). Among these, the highest prevalence for CAM visits in YH2 was among those who had visited a CAM practitioner, had self reported poor global health and had limitations due to physical or emotional health in YH1.

**Table 1:** Bivariate analysis of baseline variables and 1) future visits to CAM practitioner and 2) baseline CAM visits (N = 2429).

			CAM visits four years later (YH2)		CAM visits at baseline (YH1)	
Variables at baseline (YH1)		N	%	P-value	%	P-value
Predisposing Factors						
Visited CAM practitioner	No	2262	7.6%	<0.001*	-	
	Yes	167	24.0%		-	
Gender	Female	1298	10.9%	<0.001*	7.6%	0.117
	Male	1131	6.1%		6.0%	
Age	12	73	8.2%	0.827	5.5%	0.776
	13	789	8.9%		6.5%	
	14	903	8.5%		6.6%	
	15	542	8.1%		7.6%	
	16	122	11.5%		9.0%	
Smoked cigarettes daily	No	2338	8.6%	0.678	6.8%	0.754
	Yes	91	9.9%		7.7%	
Was active in sports	No	837	10.2%	0.062	7.6%	0.276
	Yes	1592	7.9%		6.5%	
Had been intoxicated	No	1795	8.7%	0.990	6.7%	0.533
	Yes	634	8.7%		7.4%	
Medical Need Factors						
Self reported global health	Very good	815	6.9%	0.013*	7.5%	0.387
	Good	1392	9.1%		6.5%	
	Fair	177	11.9%		6.8%	
	Poor	11	27.3%		18.2%	
Limitation due to physical health	No	2305	8.2%	<0.001*	6.4%	<0.001*
	Yes	124	17.7%		16.1%	
Limitation due to psychological health	No	2351	8.4%	0.003*	6.7%	0.035*
	Yes	78	17.9%		12.8%	
Was content with life	Yes	2090	8.2%	0.028*	6.4%	0.013*
	No	339	11.8%		10.0%	
Felt lonely	No	1841	7.9%	0.019*	6.5%	0.218
	Yes	588	11.1%		8.0%	
Had recent complaint(s)	No	157	3.8%	0.025*	4.5%	0.216
	Yes	2272	9.0%		7.0%	
Had disease(s)	No	699	5.7%	0.001*	4.4%	0.003*
	Yes	1730	9.9%		7.9%	
Used conventional medicine(s)	No	785	5.2%	<0.001*	5.1%	0.017*
	Yes	1644	10.3%		7.7%	
Visited physician	No	1167	7.3%	0.018*	4.4%	<0.001*
	Yes	1262	10.0%		9.2%	
Visited psychologist	No	2387	8.5%	0.064	6.4%	<0.001*
	Yes	42	16.7%		33.3%	

P values are from Pearson chi square test.

*p-value <0.05.

To identify the variables predicting visits to a CAM practitioner four years later, a multivariable logistic regression model was used (table 2). It correctly predicted 91.4% of all cases. When controlling for all the other variables in the model, the only variables significantly predicting increased odds that an adolescent would become a CAM visitor (p<0.05), was having visited a CAM practitioner (Adjusted Odds Ratio – adjOR 3.3 (2.2–5.0) and having used one or more of a range of conventional medicines (adjOR 1.6, 1.1–2.3). Being a male predicted reduced odds of visiting a CAM practitioner in the future (adjOR -0.6, 0.4–0.8). Omitting those who had visited a CAM practitioner in YH1 from the multivariable logistic regression analysis did not change the main findings.

**Table 2:** Multivariable logistic regression models of baseline variables 1) predicting CAM visits four years later and 2) associated with CAM visits at baseline (N = 2395).

	Prediction		Association	
	CAM visits four years later (YH2)		CAM visit at baseline (YH1)	
Baseline variables (YH1)	AdjOR (95%CI)	P-Value	AdjOR (95%CI)	P-Value
Predisposing Factors				
Visited CAM practitioner	3.28 (2.17–4.96)*	<0.001	-	
Male	0.60 (0.44–0.82)*	0.002	0.81 (0.58–1.14)	0.223
Age				
- 12 years	Ref.		Ref.	
- 13 years	1.28 (0.49–3.33)	0.616	1.05 (0.36–3.05)	0.931
- 14 years	1.16 (0.45–3.03)	0.758	0.95 (0.33–2.77)	0.932
- 15 years	1.06 (0.39–2.88)	0.903	1.09 (0.37–3.26)	0.872
- 16 years	1.36 (0.44–4.23)	0.590	1.25 (0.36–4.35)	0.727
Smoked cigarettes daily	0.97 (0.44–2.12)	0.933	0.99 (0.41–2.39)	0.985
Was active in sports	0.83 (0.61–1.14)	0.248	0.86 (0.61–1.22)	0.399
Had been intoxicated	0.85 (0.57–1.27)	0.431	0.83 (0.54–1.28)	0.399
Medical Need Factors				
Self reported global health				
- Very good	Ref.		Ref.	
- Good	1.12 (0.79–1.59)	0.507	0.69 (0.48–0.99)*	0.044
- Fair	1.32 (0.74–2.38)	0.349	0.47 (0.23–0.96)*	0.038
- Poor	2.78 (0.54–14.28)	0.220	0.87 (0.14–5.51)	0.883
Limitation due to physical health	1.48 (0.86–2.56)	0.158	2.28 (1.30–4.01)*	0.004
Limitation due to psychological health	1.53 (0.76–3.07)	0.231	0.84 (0.37–1.94)	0.691
Was content with life	0.97 (0.64–1.49)	0.902	1.55 (0.98–2.47)	0.063
Felt lonely	1.11 (0.79–1.57)	0.545	0.94 (0.63–1.40)	0.763
Had recent complaint(s)	1.78 (0.71–4.48)	0.222	1.08 (0.48-2.42)	0.854
Had disease(s)	1.36 (0.93–1.99)	0.108	1.60 (1.05–2.45)*	0.029
Used medicine(s)	1.59 (1.08–2.33)*	0.018	1.15 (0.77–1.71)	0.495
Visited physician	1.08 (0.80–1.47)	0.615	1.94 (1.36–2.76)*	<0.001
Visited psychologist	1.16 (0.45–2.98)	0.753	6.84 (3.28–14.29)*	<0.001

AdjOR – adjusted Odds Ratio - all variables in the model adjusted for each other.

*P-value < 0.05.

To further identify the underlying variables that predict future CAM visits in adolescents, those variables making up the “recent complaints”, “diseases”, and “conventional medicines” were tested in a bivariate analysis. Those with a statistical significant relationship with future CAM visits were then entered into a mulitivariable logistic regression model together with the significant variables from the larger model. In the final model (table 3), the variables predicting increased visits to a CAM practitioner four years later (p<0.05) was visiting a CAM practitioner (adjOR 3.4), having had musculoskeletal pain (adjOR 1.5), had migraine (adjOR 2.3) or other disease that lasted more than three months (adjOR 2.1) or using asthma medicines (adjOR 1.8). Being a male predicted decreased visits (adjOR 0.6).

**Table 3:** The final multivariable logistic regression models of baseline variables 1) predicting CAM visits four years later and 2) associated with CAM visits at baseline (N = 2429).

	Prediction		Association	
	CAM visits four years later (YH2)		CAM visit at baseline (YH1)	
Baseline variables (YH1)	AdjOR (95%CI)	P-Value	AdjOR (95%CI)	P-Value
Visited CAM practitioner	3.43 (2.31–5.11)	<0.001		
Male	0.57 (0.42–0.77)	<0.001		
Musculoskeletal pain	1.45 (1.08–1.94)	0.012		
Migraine	2.29 (1.19–4.39)	0.013		
Other disease that lasted more than three months	2.06 (1.12–3.77)	0.020		
Asthma medicines	1.83 (1.20–2.78)	0.005		
Limitation due to physical health			1.94 (1.13–3.34)	0.016
Visited physician			1.87 (1.32–2.65)	<0.001
Visited psychologist			6.42 (3.19–12.92)	<0.001
Itchy-watery eyes accompanying sneezing, runny or blocked nose without a cold or the flu			1.92 (1.24–2.96)	0.003
Otitis			1.60 (1.08–2.38)	0.020
Eczema			1.42 (1.03–1.97)	0.035

AdjOR – adjusted Odds Ratio - all variables in the model adjusted for each other.

### Association

To look at the differences in prediction and association, the association with CAM visits in YH1 was investigated (table 1). The bivariate analysis showed that in YH1 the highest prevalence for CAM visits was among those who had poor self reported global health, limitations due to physical health, and who had visited a psychologist.

The multivariable logistic regression showed that having good (adjOR 0.7) or fair (adjOR 0.5) self reported global health was associated with reduced odds of CAM visits in YH1 (table 2). Limitations due to physical health (adjOR 2.3), having had one of a range of diseases (adjOR 1.6), and having visited a physician (adjOR 1.9) or a psychologist (adjOR 6.8) increased the odds. The final multivariable model (table 3), showed that having visited a psychologist (adjOR 6.4) or a physician (adjOR 1.9), experiencing limitations due to physical health (adj OR 1.9) or having allergic conjunctivitis (adjOR 1.9), otitis (adjOR 1.6) or eczema (adjOR 1.4) was associated with increased odds of visits to a CAM practitioner.

### Prediction vs. association

None of the variables in YH1 that significantly predicted CAM visits in YH2 were associated with CAM visits in YH1 in either the full model (table 2) or in the final model (table 3).

## Discussion

Future visits to a CAM practitioner were predicted by being female, having visited a CAM practitioner previously, having had musculoskeletal pain, use of asthma medicines, experience of migraine or another disease lasting more than three months. None of the variables predicting future CAM visits were associated with CAM visits in the same year.

### Strengths and limitations

The main strengths of this study are that a significant proportion of those invited to enter the study did so and the stability of the population in this part of Norway allowed for a very high follow up rate four years later. The size of the study allowed for analyses with a large number of independent variables and also analyses involving both prediction and association.

However, there are some limitations. The question about visiting a CAM practitioner only mentioned a limited number of modalities and it is likely that the prevalence reported here could be on the low side due to reduced recall. Furthermore, self medication with CAM products and use of CAM self help practices was not included in the questionnaire. The utilisation of these types of CAM is known to be at least as extensive as visit to practitioners.

### Prevalence

The observed prevalence of 8.7% visiting a CAM practitioner in YH2 (2001) is similar to that for adults in the same population (9.4%) [14]. It was higher than the 2.0% visiting a CAM practitioner in USA in a 1996 study [5], but lower than the 23% visiting practitioners in San Diego, USA in 2001 [15]. We observed a significant increase over four years. This could indicate that visits to CAM practitioners have become more common, but could also reflect the general increase in the proportion of adolescents experiencing health problems as they get older.

### Predictions

As expected [16], previous visits to a CAM practitioner was the strongest predictor of future visits. Although only one in five had visited four years earlier, others could have visited in other periods suggesting the previous visits could be an even stronger predictor than observed in this study. As children's health care use is strongly related to parents' health care use [17], [18], it is likely that older adolescents continue to use the health service utilisation pattern that their parents exhibit [7], [19]. However, it has been found that more than half of CAM visitors among homeless youth were referred by friends [20].

According to the SBM, both demographic factors and medical need factors should be associated with CAM use. Indeed, although it is known from cross sectional surveys that CAM practitioners are visited by people with chronic conditions [21], socio demographic variables usually play an equally important role [14]. This is evident in this study, where none of the chronic diseases mentioned were associated with current CAM visits, but having experienced musculoskeletal pain or chronic disease (migraine, use of asthma medicines and other diseases lasting longer than three months) predicted future CAM visits. A Canadian study found that if parents perceived conventional medications to be unhelpful it predicted longer CAM use in their children [7]. Furthermore, with chronic complaints it might be that the patients have used conventional medicine with limited benefit, thus triggering visits to CAM practitioners [22].

A consistent finding in studies on CAM utilisation is that females are more frequent users. This study also concludes that being a male adolescent predicted reduced odds of visiting a CAM practitioner in the future. However, gender was not associated with CAM visits the same year, a finding in line with other studies [2], [15].

### Difference between prediction and association

Overall, medical need factors were associated with CAM use both cross-sectionally and longitudinally. However, the somewhat surprising finding in this study was that none of the specific variables predicting future CAM visit were associated with CAM visits the same year. Longitudinal studies can be used to make causal inferences while cross sectional surveys never provide rigorous answers about causal inferences. Thus, the associations derived from surveys only give an indication of a possible relationship between the dependent and independent variables. Our study clearly reinforces the notion that time is a vital variable. The environment in which a visit to a specific health care provider may occur can be identified several years prior to the visit, but it is the more immediate experiences of ill health that influence the decision to actually seek treatment. Therefore, we would expect a difference between the results of longitudinal and cross sectional studies in relation to these associations. We would also encourage the use of fine-grained longitudinal approaches (such as diary methods) to develop a more nuanced understanding of the changing relationship between medical need and healthcare utilization over time.

In the present study we did not ask directly why people sought a particular health care provider but used unrelated questions (e.g. questions on diseases were included in the questionnaire independently of the questions relating to CAM visits). Thus, having a disease and visiting a CAM practitioner may be unrelated. Future studies would benefit from including the question “what are the reason(s) for your visit?”. The specific question used could be derived from previous qualitative studies [22].

### Conclusion

We can conclude from this study that future visits to a CAM practitioner are predicted by both predisposing factors (being female, having visited a CAM practitioner previously) and medical need factors (having had musculoskeletal pain, migraine, used asthma medicines or experienced another disease lasting more than three months). None of the specific variables associated with CAM visits were predictive for CAM visits four years later.
